# The Editor's Letter-Box

**Published:** 1916-05-13

**Authors:** Félicie Norton

**Affiliations:** 2nd Birmingham War Hospital, Northfield


### THE EDITOR'S LETTER-BOX.

### Nurse-Training, Small Hospitals, and the College of Nursing.

*To the Editor of* The Hospital.

Sir,—In a recent issue of your paper you invited sisters who had had experience to express their views upon points connected with hospital administration which could perhaps later be brought to the notice of the College of Nursing. Having held the post of sister in several hospitals, and being very keen upon the training of nurses and the raising of the status of the profession, I hope I may be permitted to put forward the following views:—

Among the needed reforms appear to me to be:

1. *Affiliation Between the Various Hospitals.*—This question is too big a one to enter into in detail here, but I will quote two examples: (*a*) A woman who enters a "cottage" or women's hospital because she is obliged to do something until she is old enough to enter a training school, and has satisfactorily completed a year in such an institution, should, when she goes to a general hospital, be treated *not* as a "raw" probationer, but be placed immediately upon the same footing as those nurses who have spent, say, six months in their training school. (*b*) The nurse training in a provincial hospital of between sixty and one hundred beds should have the advantage—towards the end of her three years—of spending three months either in a very large provincial hospital or in one of the London ones. She would thus obtain the benefits of both institutions. I might add: (*c*) If a four years' curriculum be decided upon it should include three months' maternity and three months' fever work, instruction in massage, and perhaps some mental experience, this being optional.

(2) *Provision of Time for Study.*—This I consider to be urgently needed. Under present arrangements nurses attend lectures once or twice a week, and in between time theory is left to look after itself. It follows that either study is neglected or off-duty time is sacrificed. When nurses give up part of their off-duty to study that time can hardly be looked upon as a mental rest. It frequently happens that near the examination time studious nurses jeopardise their health by concentration on technical books during almost all their off-duty time. They return to the wards as tired as they left them. Nurses who are not particularly keen on study use their off-duty time solely for rest and recreation (and this is as it should be); but they have to make up for lost time by "cramming" just before the examination, or failing to satisfy the examiners. Work that has been "crammed" cannot be of any lasting benefit to the probationer.

May I, before concluding, offer you my sincere congratulations upon the foundation of the College of Nursing, which you have done much to bring about?—Yours faithfully, Félicie Norton.

2nd Birmingham War Hospital, Northfield,
May 8, 1916.

